# Advancing spatial-omics through Pyxis, vendor-neutral software for ion mobility mass spectrometry imaging data analysis

**DOI:** 10.1007/s00216-025-06281-5

**Published:** 2025-12-20

**Authors:** Sara Tortorella, Sebastian Bessler, Giuseppe Arturi, Giulia Sorbi, Jens Soltwisch, Gabriele Cruciani

**Affiliations:** 1 Mass Analytica SL, Av. Cerdanyola 92-94, 08173 Sant Cugat del Vallés, Spain; 2 Institute of Hygiene, University of Münster, Robert-Koch-Str. 51, 48149 Münster, Germany; 3 Molecular Horizon Srl, Via Montelino 30, 06084 Bettona, Perugia Italy; 4 Department of Chemistry, Biology and Biotechnology, University of Perugia, Via Elce Di Sotto 8, 06123 Perugia, Italy

**Keywords:** Mass spectrometry imaging, Ion mobility, Software and data analysis tools, Metabolomics, Chemometrics

## Abstract

Mass spectrometry imaging (MSI) is a powerful analytical technique suited for simultaneously measuring and assigning functional roles of multiple analytes directly from intact tissue sections. MSI acquisitions often lead to highly complex datasets with numerous isobaric species. Ion mobility (IM) spectrometry crucially helps to unravel these datasets by providing an orthogonal separation thus supplementing the lack of chromatographic separation in MSI. However, the rich, multidimensional data produced by IM-MSI investigations, combined with the lack of comprehensive software solutions that support the entire data analysis workflow, poses a major challenge preventing IM-MSI full exploitation. Here, we discuss the benefits and challenges of IM-MSI data analysis in metabolomic applications. Finally, Pyxis, a novel, vendor-neutral IT solution for IM-MSI data analysis, is introduced and its capabilities demonstrated on mouse kidney tissue and THP-1 monocytes data.

**Graphical Abstract:**

**Figure:**
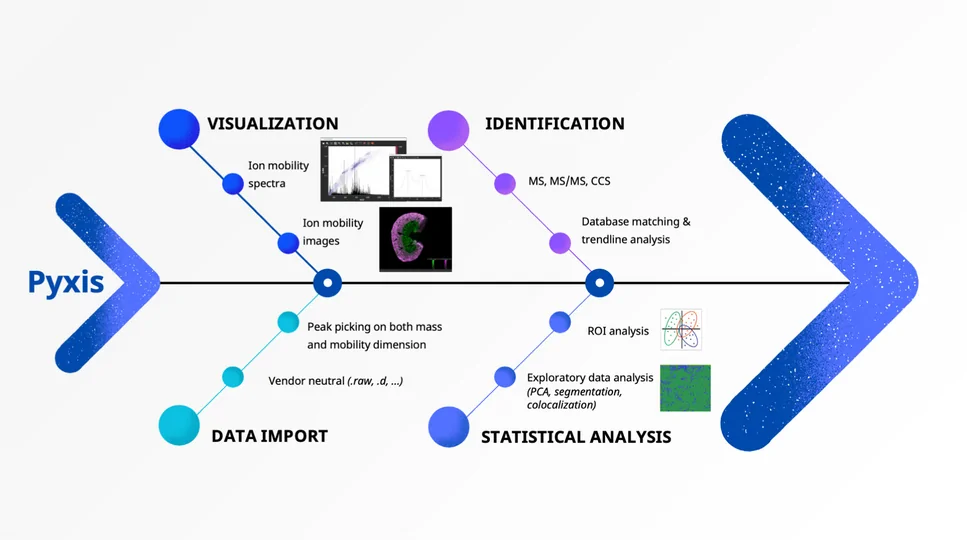

**Supplementary Information:**

The online version contains supplementary material available at https://doi.org/10.1007/s00216-025-06281-5.

## Introduction

Mass spectrometry imaging (MSI) is a powerful analytical technique that combines the principles of mass spectrometry and spatial imaging, allowing researchers to generate detailed molecular maps of complex samples, such as tissues, cells, and other surfaces. MSI can be used to study the distribution of drugs, metabolites [1], lipids [2], peptides [3] and glycans [4] and has a wide range of applications in various fields. In medical research, MSI can provide insights into disease mechanisms, therapeutic treatments, and biomarkers’ discovery [5–10]. In pharmacology, MSI helps researchers understand how drugs are distributed within tissues and organs, aiding in drug development (i.e., early toxicity and efficacy assessment) and optimization [11–13]. In environmental science, MSI has recently proved crucial in the analysis of contaminants, pollutants, and biomolecules in environmental samples [14]. In forensics, MSI can be applied to study the distribution of chemicals and compounds, helping to gather evidence and understand crime scenes [15–17].

Various desorption/ionization techniques are used for MSI, each one with its own advantages and pitfalls, with matrix-assisted laser desorption/ionization (MALDI [18]), desorption electrospray ionization (DESI [19]), and secondary ion mass spectrometry (SIMS [20]) being the most popular.

A substantial common challenge in MSI experiments is effectively capturing the molecular complexity of tissue and identifying the numerous chemical species therein. Inherent to MSI analysis, minute amounts of a complex mixture of analyte molecules are directly sampled from the surface of a sample such as a tissue section. Naturally, this prohibits the use of separation techniques such as liquid chromatography (LC) and largely hampers the use of tandem-MS. Typically, chemical identification in MSI has therefore relied on a combination of high mass accuracy and resolving power for tentative assignment based on accurate mass alone.

Coupling the ion source with high mass resolving power analyzers (e.g., Orbitraps and Fourier-transform based-ion trap instruments) proved successful in the resolution and identification of isobaric ion species [21–24]. Nevertheless, it is important to note that high mass accuracy alone is not sufficient for distinguishing structural isomers in complex biological samples. For instance, in lipidomic investigations, high mass resolution enables confident assignment of lipid classes and sum compositions, while the identification of individual fatty acyl chains, needed for a complete elucidation of lipid biochemistry, is only achievable with MS/MS and/or chemical derivatisation [25, 26].

To address this limitation, parallel MSI and liquid chromatography (LC)-MS/MS experiments can be conducted. This can be achieved by enriching MSI data with offline LC-MS/MS of tissue extracts, by laser micro-dissection coupled with LC-MS/MS, or by collecting fragmentation data directly from the tissue. Each strategy has its advantages and limitations, as nicely overviewed by Claes et al. [27]. Direct tissue MS/MS, although offering high spatial fidelity in tandem MSI experiments, is constrained by the number of species that can be fragmented from a single pixel, along with a long time for acquisitions [28]. Recently, DESI-MSI has been coupled to triple quadrupole MS analysis that allows for the analysis of up to 30 precursors per pixel by rapid switching of precursor window [29]. Here, isobaric lipid ion species could be separated by choosing the right multiple-reaction-monitoring (MRM) transition for each. Alternatively, on-tissue chemical derivatization and enzymatic digestion have been developed as targeted methods to selectively increase ion yields for a specific class of analyte or deplete signal intensity of suppressors [30–32].

**Fig. 1:**
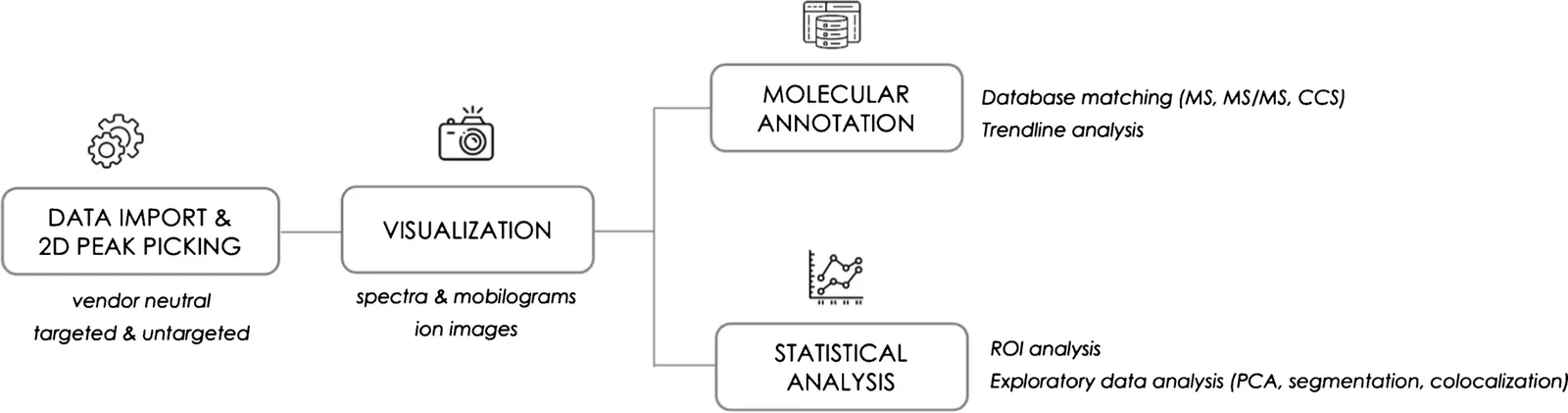
Pyxis data analysis schematization

More recently, the application of ion mobility spectrometry (IMS) available through different technical realizations such as drift tube IMS (DTIMS), traveling wave IMS (TWIMS), and trapped IMS (TIMS) has proven to be a valuable tool for improving compound annotation in MSI analyses. In contrast to chromatographic separation, it is based on the interaction between gaseous ions and a background gas and provides an additional molecular descriptor, the collision cross section (CCS [33, 34]) that is orthogonal to the mass-to-charge ratio (m/z) value. Because of its working mechanism, it can be conducted between the generation of ions inside the ion source and the mass spectrometric analysis. Consequently, it is amenable to MSI and can serve to enhance specificity, improve molecular coverage, and aid in molecular identification, all while maintaining acquisition times in the typical range for MSI. For instance, the combination of MALDI-2 with TIMS allowed the separation of numerous isomeric/isobaric ion species, unravelling the complex lipidomic composition of the tissues [35]. Ion mobility MSI (IM-MSI) has proven invaluable in the structural analysis of peptides, lipids, and in proteomics applications [36–39], demonstrating that feature discovery within the 2D m/z-ion mobility domain, as opposed to exclusively focusing on the m/z domain, results in a significant increase in the number of identified features and provides a second independent variable to characterize each feature. However, adding an additional independent dimension translates into an increment in the size and complexity of the obtained dataset. Efficiently processing these extensive, multidimensional datasets demands substantial runtime and memory resources. Currently, one of the primary challenges lies in the absence of vendor-neutral and comprehensive software solutions that cover the entire workflow essential for a thorough IM-MSI data analysis, which could be simplified as (1) pre-processing; (2) spectra and image visualization; (3) data mining; and (4) identification, all within the 2D m/z-ion mobility domain (Fig. 1). As reported in a recent review by Ross et al. [40], the majority of published IM-MSI datasets have been processed using vendor-provided software: High Definition Imaging (Waters, Wilmslow, UK) for TWIMS-MS instruments or SCiLS Lab (Bruker Daltonics, Bremen, DE) for data generated on TIMS-MS platforms. A smaller yet significant number of studies have relied on custom data analysis pipelines, often developed in MATLAB or Python, which only partially address the full IM-MSI data analysis workflow [40].

To tackle this challenge, we developed Pyxis. Pyxis is a vendor-neutral IT platform tailored for IM-MSI targeted and untargeted data analysis. The Pyxis data analysis workflow, illustrated in Fig. 1, comprises all the essential steps for IM-MSI data analysis, including raw data processing, histological co-registration, image and mobilogram visualization, a diverse range of univariate and multivariate image and spectral analysis tools, segmentation, and co-localization analysis. Notably, Pyxis also introduces an integration of automated metabolite identification (leveraging both high mass accuracy, MS/MS, and CCS data), significantly boosting biochemical data interpretation.

After a detailed description of how pre-processing, visualization, data mining, and identification are executed within Pyxis, we will present two case studies focusing on IM-MSI metabolomics investigation of tissues and single cell datasets.

## Materials and methods

### Sample preparation

Kidney tissue was obtained from a healthy 16-week-old male C57BL/6J mouse sourced from the central animal breeding facility of the University Hospital Münster. Animal husbandry and organ harvesting were conducted according to protocols approved by the local ethical review committees. After tissue harvest, the kidney was embedded in M1 embedding medium and snap frozen in liquid nitrogen. Samples were stored at −80 °C until further processing. Sixteen-micrometer-thick tissue sections were produced with a cryomicrotome and thaw-mounted onto glass slides. Before matrix application, the tissue was dried under a gentle stream of dry nitrogen gas.

THP-1 ACC 16 cells (DSMZ) were cultured in RPMI 1640 Medium (Lonza), supplemented with 1 mM sodium pyruvate, 10% fetal bovine serum, and 2 mM L-glutamine. Cells were sewn in Millicell EZ Slide 8-well chamber slides (Sigma-Aldrich). Differentiation was induced by exposure to 20 ng/mL phorbol 12-myristate 13-acetate (PMA) for 24 h followed by a 3-day rest phase in PMA-free medium. THP-1 derived macrophages were polarized using IL-4 and IL-13 (20 ng/mL) for 48 h. Before matrix application, cells were fixed using 4% formaldehyde in PBS for 5 min, washed with 150 mM ammonium acetate, and then air-dried at room temperature.

Samples were sublimation coated with 2,5-dihydroxyacetophenone (2,5-DHAP) prior to MALDI-MSI. Coating was performed with a home-built sublimation device as described by Bien et al. [41]. Briefly, 1.5 mL of 20 mg/mL 2,5-DHAP in acetone was evaporated and heated to 130 °C. The sample was maintained at 4 °C. Sublimation was conducted for 10 min at approximately 5*10^–3^ mbar.

### Data acquisition

Samples were analyzed using a timsTOF flex MALDI-2 instrument (Bruker, DE). The kidney tissue was measured with MALDI-2 post-ionization using a pixel size of 20 µm, 100 laser shots per pixel, a primary laser power of 80%, and an inter laser delay of 10 µs. For TIMS separation a ramp time of 100 ms with a 1/k0 range of 0.8 to 1.8 was used. The m/z acquisition range was 300–1500. THP-1 cells were analyzed with a pixel size of 15 µm, 54 laser shots per pixel, a primary laser power of 80%, and an inter laser delay of 10 µs. TIMS separation was performed with a ramp time of 100 ms and a 1/k0 range of 0.7–1.8, with an m/z range of 140–1500. Prior to all analyses, the TIMS separation was calibrated using the ESI-L low molecular weight TuneMix.

## Data analysis workflow

The data analysis workflow consists of the following steps: pre-processing, spectra and image visualization, statistical analysis (further divided into exploratory and region of interest analysis), and identification. Given the computational complexity inherent in such datasets, all algorithms have been fine-tuned and parallelized to fully harness the capabilities of modern multi-core hardware architectures, thus optimizing calculation performances. A detailed description of the single steps is provided in the next paragraphs.

### Pre-processing

While the MSI community has agreed on an open vendor-neutral format for regular MSI datasets (imzML [42]), MSI data which contains additional IMS information is, to date, only available in proprietary, vendor-defined formats. Pyxis was therefore designed to directly import and process data acquired with the IM-MSI instrumentations commercially available to date. These include the timsTOF fleX MALDI(−2) (Bruker, DE), Synapt, and Cyclic TOFMS DESI (Waters) platforms. Additional data formats could be added in the future, as the software is designed to be vendor neutral.

Pyxis allows to analyze data either in a targeted fashion, that is, by defining range(s) of m/z and/or mobilities values of interest, or following an untargeted approach. Likewise, these targeted and untargeted approaches extend to the spatial domain: the software allows to focus on the entire area of the sample under investigation (untargeted) or on a subsection of it (targeted). Then, the peak detection on the 2D m/z-ion mobility domain comprises the following algorithms: Savistsky-Golay smoothing, baseline correction, and peak picking (SI, section S1). The data is first picked in the mass spectra domain; afterwards, each m/z peak is peak picked in the mobility domain (also known as extracted ion mobilogram [34]).

After the completion of the peak detection step, the dataset building algorithm is executed. This algorithm enables user-defined filtering by frequency, absolute intensity, spatial chaos [43], and to perform the isotope clustering [44, 45].

The whole process produces a list of features with a distinct m/z-mobility (m/z@mobility) combination, to be used for visualization and as input for the exploratory data analysis algorithms. Nevertheless, raw data could still be accessed both at the spectra inspection and image visualization levels. More details on the pre-processing algorithm are available in SI, Section S1 (Figures S1.1-S1.4). 

### Visualization

The mobility dimension is taken into account both at the spectra and image visualization level. 2D plots (m/z *vs* mobility) are available for both single pixel and average sample inspection. Their inspection allows to identify potential isobar species with different mobility values, one of the primary goals in IM-MSI data analysis. Normalization, smoothing, denoising, and hotspot removal [46] algorithms are available to optimize the feature (i.e., m/z@mobility) intensity distribution visualization. A semi-automatic co-registration process, or the automatic reading of “reference points,” allows for the co-registration of multiple images, such as high-resolution histological, fluorescence, or optical images, with MS images, thereby enhancing biochemical interpretation. Transparency and color scales can be easily adjusted, enhancing the visualization of both single and multiple features as overlay images [47]. All of the images can be exported in publication-ready quality or added to an internal report for future inspection. More details on the visualization algorithms are available in SI, Section S2 and Figure S2.1.

### Exploratory Data Analysis (EDA)

In EDA, the samples are analyzed without any a priori knowledge. Several algorithms are available in Pyxis, including spatial segmentation [48, 49] principal component analysis (PCA [50, 51]), and co-localization [51] algorithms. The input of these algorithms is the entire set of m/z@mobility features obtained at the end of the pre-processing step: without any external bias, these algorithms look for feature similarities and group the pixels according to it. Co-localization analysis can be applied to specific feature distribution of interest, returning a list of m/z@mobility values exhibiting spatial distributions similar to the input, where the similarity is quantified using a Pearson correlation coefficient [52, 53]. Additionally, the isobar scout algorithm was designed and implemented to automatically find features showing the same m/z value (within a custom tolerance) but with different mobility values and with different spatial distribution. The algorithm also works in the opposite direction, that is, grouping the features showing the same mobilities (within a custom tolerance) but with different m/z values. In this way, we streamline one of the crucial needs of the IMS-MSI community: using the mobility dimension to separate true isobaric and near-isobaric species, which eventually show different distribution on tissue and were previously, erroneously, identified as single species [34, 35]. A detailed description of the isobar scout algorithm is available in SI, Section S3.

### Regions of Interest (ROIs) Analysis

Frequently guided by histology, specific tissue areas referred to as “regions-of-interest” (ROIs) are defined. Guided by prior knowledge or expert inputs, these ROIs can be manually created or imported from software used for annotation by expert pathologists, such as QuPath [54] and Pannoramic Viewer [55]. Once the ROIs have been defined, the same multivariate data analysis tools and plots previously described [56] are available for identifying discriminant features. The use of multivariate analysis [57, 58] has proven valuable in supporting MSI investigations, especially in the metabolomics domain, offering a robust asset for revealing specific molecular changes across samples [59–64]. Moreover, Pyxis provides various unsupervised methods, such as PCA [50, 51], and supervised algorithms like partial least-squares discriminant analysis (PLS-DA [65, 66]). Instead of opting for simplicity at the cost of transparency, with an approach also known as “black-box,” Pyxis includes user-friendly advanced tools that facilitate the visualization, interpretation, and assessment of the statistical analysis outcomes, including evaluating robustness and efficacy (e.g., spurious variables, statistical artifacts).

### Identification

The use of ion mobility separation holds great potential for increasing the number of molecular identifications in MSI investigations. Pyxis takes into account all the experimental information that could be collected in an IM-MSI experiment (m/z, MS/MS, CCS) to propose a putative structural assignment of the feature of interest. As a first step, mobilities are converted into CCS through the vendor software development kit (SDK). For TIMS data, mobility (i.e., 1/k0) values are converted to CCS (in Å^2^) using the Mason-Shamp equation [67]. For TWIMS data, a direct extraction of CCS values from experimental parameters is not possible and a calibration curve compiled using well-defined standards is needed. When available, the software will automatically use the calibration curve to convert mobility into CCS values.

Then, the identification workflow is analogous to the one outlined in our earlier MSI solution [56], with the exception that in Pyxis the identification score is also weighed on the CCS score, comparing theoretical CCS values with experimental data (S4 in SI). Custom adducts can be specified, which also enables addressing case studies where functionalization strategies have been adopted. Option for additional MS/MS (e.g., from complementary ESI) importing allows to decide whether to consider the accurate mass of the ion and then either MS/MS only, MS/MS+CCS, or CCS only. Indeed, IMS and MS/MS have been recently successfully combined to provide structural elucidation and separation at the same time. This technique enables the analysis of 10–15 precursor m/z windows in the same run [28]. CCS can be derived from publicly available DBs or self-farming DBs [68, 69]. Most of the commonly used database in metabolomics investigations can be imported in Pyxis: HMDB [70], GNPS [71], AllCCS [68], as well as.mgf and.csv generic formats as it could be for in house databases. In case of unavailable or incomplete information provided by the chosen DB, trend line plots of m/z vs CCS values can be used to assign unknown features to a group of previously assigned features (see Fig. 4) [37]. More details on the identification algorithm are available in the SI, Section S4.

## Case studies

To demonstrate the unique capabilities for data mining provided by Pyxis, datasets from mouse kidney tissue as well as a cell culture of THP-1-derived macrophages were analyzed. Data import parameters are summarized in SI, Section S5.

### Kidney tissue data analysis

**Fig. 2:**
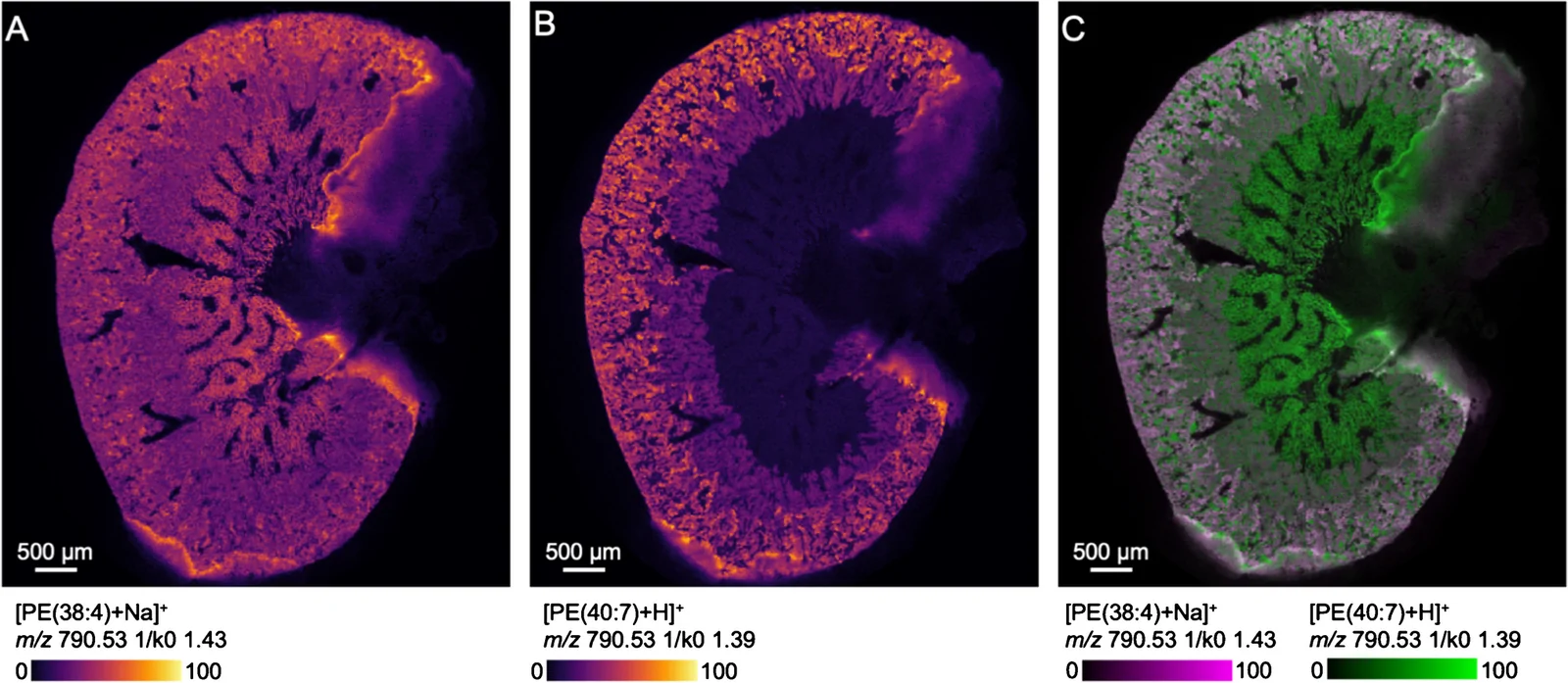
MALDI-TIMS-MSI of mouse kidney tissue. **A**, **B** MALDI-TIMS-MSI images, displayed with Inferno color scale, of the features at m/z 790.53 reveal two distinct features clearly separated by ion mobility. **C** Overlaid visualization of the two features

For the kidney tissue, a peak list with more than 150 entries was generated. Visualization of most of these signals shows little to no intensities in areas outside the tissue highlighting the capabilities of IM-MSI to increase S/N by removing background chemical noise. To explore if any isobaric species in the m/z domain were separated by ion mobility, the isobar scout algorithm (described in the previous section and in SI, section S3) was used. This led to the identification of two signals both with the m/z value of 790.53, one having a mobility (i.e., 1/k0) value of 1.42 and the other of 1.38 (Figure S5.1). Comparing this to data published by other groups, the signal at 1/k0 = 1.39 was identified as [PE(40:7)+H]^+^ and the one at 1/k0 = 1.43 as [PE(38:4)+Na]^+^ [72]. While not being true isomers, a very high mass resolution (R > 300,000) would be needed to separate these two peaks which is not feasible for most instruments or would lead to long measurement times, as discussed above. The same measurement without IMS separation would show a composite of the two images, leading to potential misinterpretations, as the two lipid signals show vastly different distributions: [PE(38:4) + Na]^+^, for example, is detected in the medulla of the kidney, whereas [PE(40:7) + H]^+^ is largely absent, suggesting distinct physiological roles (Fig. 2).

### THP-1-derived macrophages data analysis

**Fig. 3:**
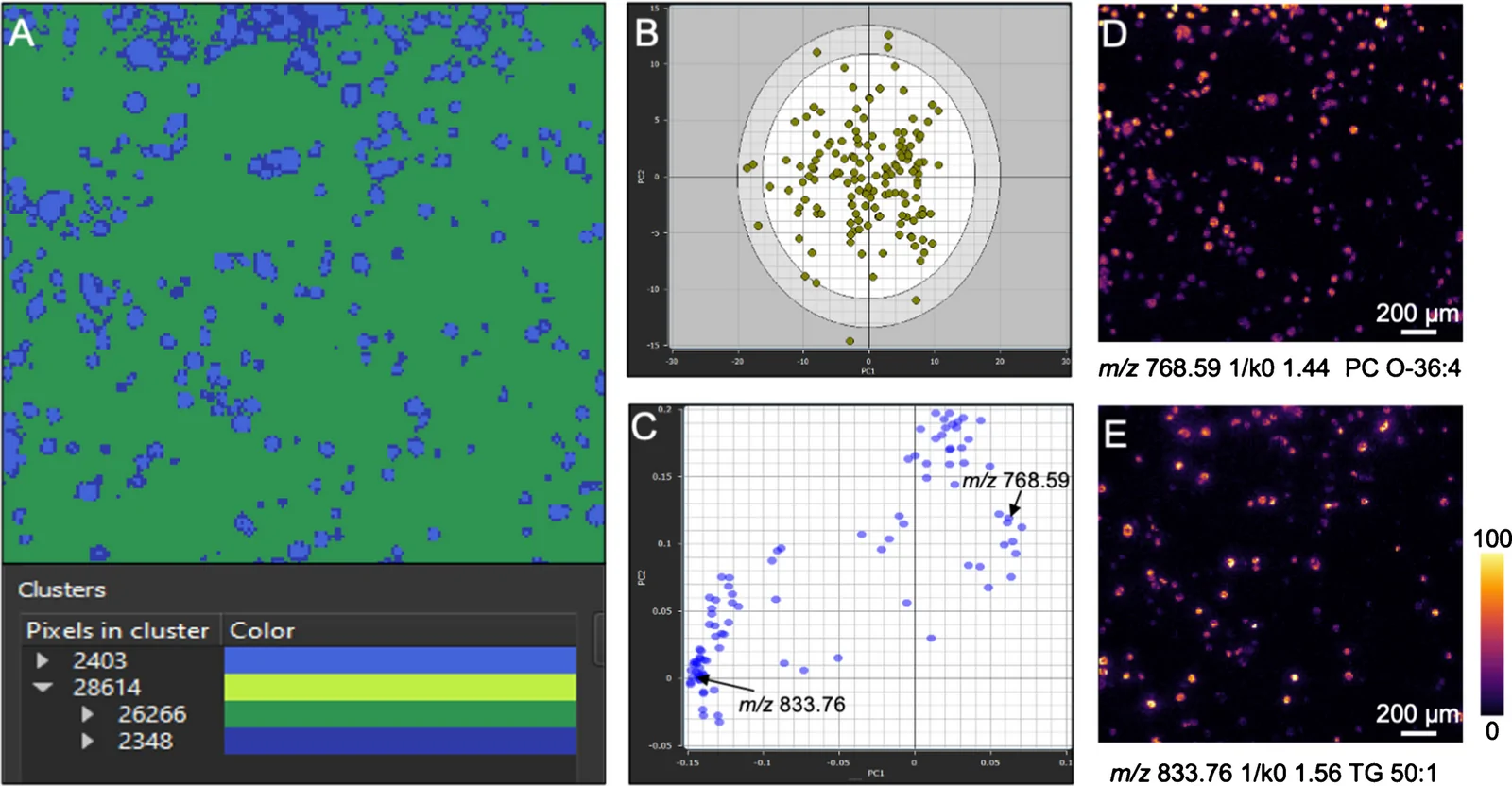
MALDI-TIMS-MSI of a cell culture of THP-1 derived macrophages. **A** Spatial clustering enables the generation of ROIs that only include single cells or small cell clusters. **B**, **C** PCA scores (**B**) and loadings (**C**) of the ROIs reveal several *m/z* values which display a high degree of heterogeneity in the cell culture. **D**, **E** MALDI-TIMS-MSI images of PE O-36:4 and TG 50:1, displayed with Inferno color scale

**Fig. 4:**
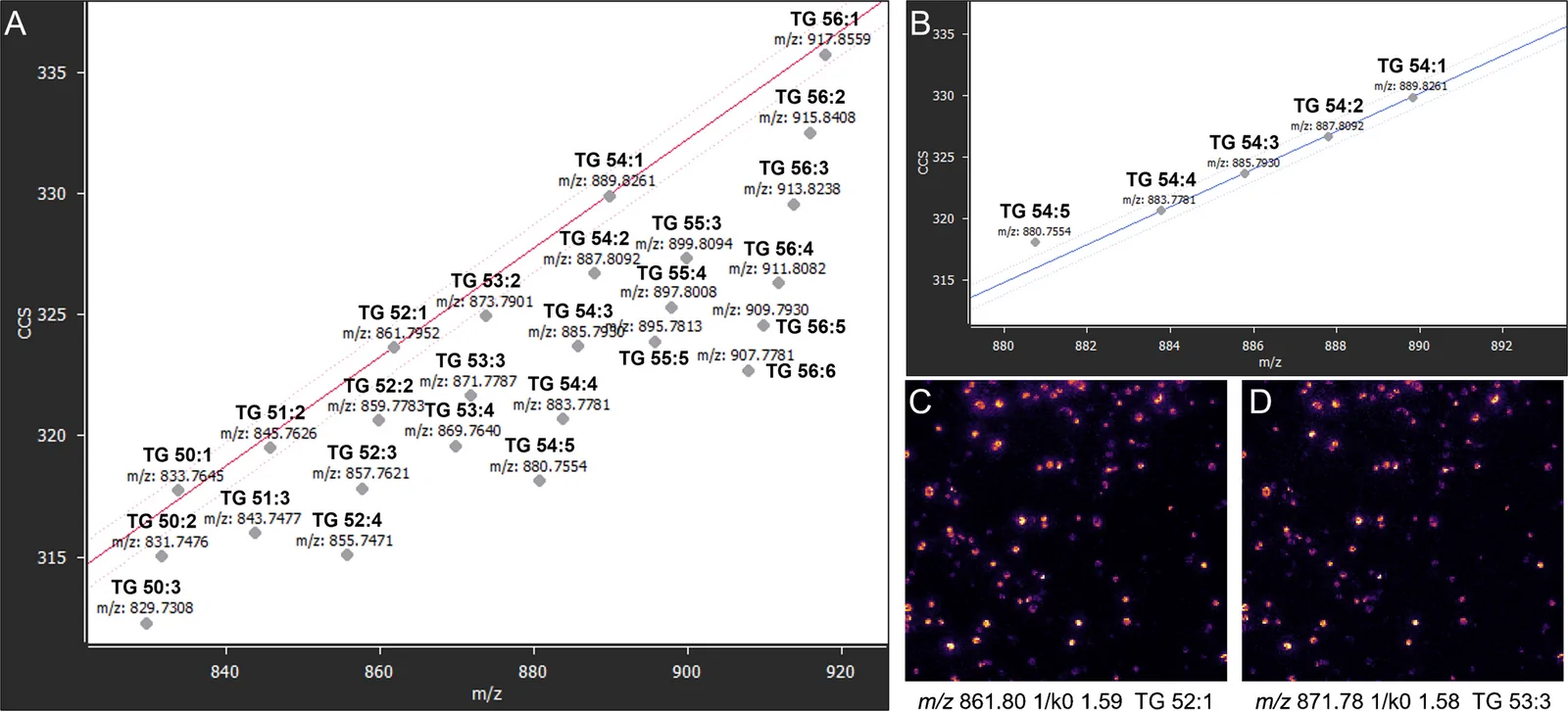
Trend line plots for triglyceride (TG) signals in THP-1 derived macrophages. **A**, **B** CCS values for TGs show a high degree of linearity for TGs with the same number of double bonds but a different number of carbons (**A**) and for TGs with the same amounts of carbons in the acyl chain but a different number of double bonds (**B**). **C**, **D** MALDI-TIMS-MSI images of TG 52:1 and TG 53:3, displayed with Inferno color scale

The second case study on THP-1-derived macrophages demonstrates the ability of Pyxis to explore single cell data and help in the assignment of m/z values. Import parameters are again summarized in SI, Section S5.2. Co-localization analysis of the highly abundant lipid signal of [PC(34:1)+H]^+^ at m/z 760.59 and 1/k0 1.43 resulted in a list of signals only originating from the cells. This list was then used to create a spatial segmentation of the dataset. Based on this segmentation, a ROI encompassing cells and excluding background was defined (Fig. 3A). This ROI was then split into separated ROIs, each containing either a single cell or a small cell cluster, using a dedicated algorithm available in Pyxis. To investigate potential heterogeneity inside the cell culture, multivariate analysis was used to differentiate groups of cells with comparable lipid profiles [73, 74]. A PCA depicted in Fig. 3B reveals a relatively homogenous clustering of these ROIs. Mining the respective loadings however results in a number of m/z values that show a strong heterogeneity throughout the cell culture separated by PC1. Using the identification module based on exact mass, a large portion of these signals were tentatively assigned as triacylglycerides (TG). Within the mobilogram, TGs with the same amounts of carbons in the acyl chain but a different number of double bonds line up on comprehensible trend lines (Fig. 4A). Similarly, trend lines can be formed for groups of TGs with the same number of double bonds but a different number of carbons (Fig. 4B). This effect can be used to corroborate tentative assignments, but may also help to identify signals which are not listed in publicly available databases. Interestingly, a group of signals at m/z 843.75, 871.78, and 899.81 can be annotated by accurate mass to be TG 51:3, TG 53:3, and TG 55:3. While all three peaks lay on a trendline, they cannot be grouped with the respective TGs with 3 double bonds that carry only even chain acyl chains. This finding points to either a false assignment of the odd chain TGs or a strong difference in the cross section of the containing odd chain acyl chain as compared to its even chain counterparts. This deviation may be introduced by an unusual position of the double bond within the chain. These hypotheses will guide future investigations.

## Conclusions

In this paper, we have presented Pyxis, a novel vendor-neutral software tailored for comprehensive ion mobility MSI data analysis. To demonstrate the capabilities of the software, we presented two case studies: MALDI-2 timsTOF data analysis of kidney tissue and single cells.

The software has been designed as a user-friendly tool, capable of streamlining IM-MSI data analysis, including pre-processing, exploratory data mining and ROI analysis, image co-registration, image and spectra visualization, and automated annotation of metabolites.

Considering the growing adoption of ion mobility MSI and the ongoing enhancements in throughput and spectral quality, the demand for advanced software tools to handle the increasing complexity of data is of utmost importance. In this context, Pyxis stands as a powerful platform, enabling researchers to effectively exploit IM-MSI datasets and the valuable (bio)chemical insights they contain.

## Supplementary Information

Below is the link to the electronic supplementary material.

Supplementary Material 1 (DOCX 7.07 MB)

## Data Availability

The data supporting the findings of this study are available from J.S. (email: Jens.Soltwisch@ukmuenster.de) upon request. Pyxis is freely available for evaluation and then reduced fees apply to non-profit research institutions. If interested, please contact https://mass-analytica.com/products/pyxis/.
