# Supplementary material for: Advancing spatial-omics through Pyxis, vendor-neutral software for ion mobility mass spectrometry imaging data analysis
Source: Anal Bioanal Chem. 2025 Dec 20;418(20):6647–56. doi: 10.1007/s00216-025-06281-5 (PMC13619667; doi:10.1007/s00216-025-06281-5)
Supplement: Supplementary file 1 [file 216_2025_6281_MOESM1_ESM.docx]

**Supporting Information**

Advancing spatial-omics through Pyxis, vendor-neutral software for ion mobility mass spectrometry imaging data analysis

Sara Tortorella,^1*^ Sebastian Bessler,^2^ Giuseppe Arturi,^1^ Giulia Sorbi,^3^ Jens Soltwisch,^2*^ Gabriele Cruciani^4^

1 Mass Analytica SL, Av. Cerdanyola 92-94, 08173 Sant Cugat del Vallés, Spain

2 University of Münster, Institute of Hygiene, Robert-Koch-Str. 51, 48149 Münster, Germany

3 Molecular Horizon Srl, Via Montelino 30, 06084 Bettona, Perugia, Italy

4 Department of Chemistry, Biology and Biotechnology, University of Perugia, Via Elce di Sotto 8, 06123 Perugia, Italy

Corresponding Author

* Sara Tortorella, sara.tortorella@mass-analytica.com

Jens Soltwisch, Jens.Soltwisch@ukmuenster.de

Summary

[S1. Data Processing Algorithms	2](#_Toc205396470)

[Figure S1.1: Dataset building workflow.	2](#_Toc205396471)

[Figure S1.2: Peak detection parameters	3](#_Toc205396472)

[Figure S1.3: Mobilogram peak detection parameters	4](#_Toc205396473)

[Figure S1.4: Dataset parameters	5](#_Toc205396474)

[S2. Visualization	5](#_Toc205396475)

[Figure S2.1: Visualization settings	6](#_Toc205396476)

[S3. Isobar scout algorithm	6](#_Toc205396477)

[S4. Identification	7](#_Toc205396478)

[S5. Untargeted data analysis of MALDI-2 timsTOF data	8](#_Toc205396479)

[S5.1: MALDI-TIMS-MSI of mouse kidney tissue import settings	8](#_Toc205396480)

[S5.2: MALDI-TIMS-MSI of THP-1 derived macrophages import settings	8](#_Toc205396481)

[Figure S5.1: MALDI-TIMS-MSI of mouse kidney tissue. Mobilogram at *m/z* 790.53 of a single pixel shows two distinct features separated by ion mobility.	9](#_Toc205396482)

S1. Data Processing Algorithms

Figure S1.1: Dataset building workflow.

During the File import, Pyxis performs the following:

A) For each frame read the mass spectra for each *mobility* scan and execute the following:
   1. **Spectra Peak detection** on profile spectra: this step is executed in profile acquisitions only. The following algorithms are executed:
      - **Smoothing of the profile spectra**: the widely used Savitzky-Golay algorithm is executed.
      - **Baseline correction**: the baseline is computed by using a linear interpolation algorithm and then subtracted.
      - **Peak picking**: a local maxima peak picking algorithm is executed by filtering out peaks below under a minimum SNR and intensity values set by the user.
   2. **Centroid spectra filtering**: a filtering can be applied to:
      - Signals: by discarding the *m/z* with intensity less than a specified threshold.
      - Acquisition area: by discarding the peaks outside mass range and/or *mobility* range.
   3. **Mobilogram peak detection**: this step is executed on mobilogram built on *m/z* found in the Spectra Peak detection step:
      - **Smoothing of the profile spectra**: the widely used Savitzky-Golay algorithm is executed.
      - **Peak picking**: a local maxima peak picking algorithm is executed by filtering out peaks below under a minimum SNR and intensity values set by the user.
   4. **Building of the average spectrum** using all the centroid data.
B) The output of the processing applied to frames are, for each frame, the list of triplet *m/z*, *mobility*, intensity.
   1. **Building of the average spectrum** using all the triplet list generated for each frame.
   2. **Selection of the most relevant peaks** from the average spectrum by using the following criteria:
      - minimum peak frequency.
      - minimum spatial chaos.
   3. **Isotope clustering**: peaks of the average spectrum are clustered together when an isotope cluster is detected. This clustering uses the following information:
      - *m/z* spacing and *mobility* spacing.
      - experimental and theoretical abundance deviation.
      - Pearson correlation among the *m/z*, *mobility* images.
      Figure S1.2: Peak detection parameters

- **Window**: points to be used during the Savitzky-Golay algorithm. When this value is increased, the smoothing is stronger.
- **Degree**: degree of the polynomial to be used. Higher-order filters do best at preserving feature heights and widths but do less smoothing on broader peaks.
- **Iterations**: number of times the Savitzky-Golay the algorithm is repeated (e.g. with iterations set to 2 every resulting spectrum is the smooth of the smooth of the corresponding original spectrum).
- **Segment size**: size in amu. of the segment to be used for the computation of baseline value (that is the median of the intensities on the segment).
- **Minimum SNR**: peaks with a SNR under this value are discarded.
- **Noise window size**: size in amu. of the window to be used to compute a local noise level (used to compute the SNR value).
- **Minimum absolute intensity**: peaks with an absolute intensity under this value are discarded.
- **Discard peaks under**: peaks with an relative intensity under this value are discarded
- **Signal filtering threshold:** peaks under this threshold are discarded and not used in the post peaks detection processing of the mass spectra. This filtering is used to save time during the import processing.
- **Discard signals under threshold for image visualization**: If checked, signals below the threshold will not be used to build the ion density images. This filtering is used to save space on disk.
- **Limit acquisition area**: If checked, signals outside the specified range of *m/z* and *mobility* are not acquired.

Figure S1.3: Mobilogram peak detection parameters

- **Window**: points to be used during the Savitzky-Golay algorithm. When this value is increased, the smoothing is stronger.
- **Degree**: degree of the polynomial to be used. Higher-order filters do best at preserving feature heights and widths but do less smoothing on broader peaks.
- **Iterations**: number of times the Savitzky-Golay the algorithm is repeated (e.g. with iterations set to 2 every resulting spectrum is the smooth of the smooth of the corresponding original spectrum).
- **Minimum SNR**: peaks with a SNR under this value are discarded.
- **Noise window size**: size in amu. of the window to be used to compute a local noise level (used to compute the SNR value).
- **Minimum absolute intensity**: peaks with an absolute intensity under this value are discarded.
- **Discard peaks under:** peaks with a relative intensity under this value are discarded
- **Discard peaks with less than N peaks**: If a discovered peak is composed of less than N points the peak is discarded

Figure S1.4: Dataset parameters

- **m/z tolerance:** used to build the average spectrum and isotopic clustering.
- **Mobility tolerance:** used to build the average spectrum and isotopic clustering.
- **Min peak frequency:** peaks that have a frequency in spectra under this threshold are discarded.
- **Min peak intensity:** peaks with an intensity under this threshold are not used to build the average spectrum.
- **Min spatial chaos:** peaks that have an *m/z*-*mobility* image with a spatial chaos under this threshold are discarded.
- **m/z to use for normalization:** *m/z* that can optionally be used for normalization purposes during dataset processing or image visualization must be declared here.

The isotopic pattern clustering was developed to consider theoretical spacing and abundance based on a series of chemical formula that are compatible with the *m/z* and *mobility* values under investigation.

- **Isotopic pattern abundance deviation:** if the deviation between experimental and theoretical abundance is above this threshold peaks are not clustered together.
- **m/z image correlation threshold:** if the Pearson correlation between the *m/z-mobility* image of the P peak and candidate P+X peak is below this threshold, peaks are not clustered together.

S2. Visualization

A number of image processing algorithms can be applied to improve image quality and account for possible spectra artefacts (i.e., hotspot).

- **Smoothing:** a linear interpolation of the pixel intensity is applied.
- **Denoising:** an **edge preserving median filter** and the relative strength can be set to:
- **None**: the denoise is not applied
- **Low**: a 3x3 window is used
- **Medium**: a 5x5 window is used
- **High**: a 7x7 window is used

Denoising can be applied to data before visualization, segmentation, PCA, *m/z* co-localization.

- **Hotspot removal:** used to enhance to contrast of the images. Two algorithms are available:
- **Histogram equalization:** Improves the contrast in an image, in order to stretch out the intensity range of the image histogram.
- **Quantile thresholding:** The intensity of the "High quantile” parameter is applied to those pixels exceeding such intensity threshold.
- **Normalization:** the intensity of every peak in every spectrum is divided by a normalization coefficient that can be:
- **TIC**: the sum of the intensities of the peaks in the same spectrum
- **RMS**: root mean square of the intensities of the peaks in the same spectrum
- **m/z**: the intensity of the peak with *m/z* specified in the Dataset section of the Pyxis processing settings

Normalization can be applied to data before visualization, segmentation, PCA, co-localization.

Figure S2.1: Visualization settings

S3. Isobar scout algorithm

**1. Group features showing the same m/z within the specified tolerance:**

- The preferred list entries are sorted by m/z value.
- For each m/z group, the first entry is considered as the m/z reference value (*mzRef,* 340.2925 for GROUP 1). An m/z group contains all the entries with m/z within the tolerance range:

m/z >= (*mzRef -* tolerance) && m/z <= *mzRef* + tolerance

**2. Group features showing the same mobility within the specified tolerance:**

Each m/z group is split into multiple groups according to the mobility (M) value and the given tolerance.

- The preferred list entries are sorted by M.
- For each mobility group, the first entry is considered as the M reference value (*MRef,* 0.9360 for the GROUP 1). A mobility group contains all the entries with mobility values included in the tolerance range:

M >= (*MRef -* tolerance) && M <= (*MRef* + tolerance)

- For each group, the entry with the best spatial chaos is chosen.

**3. Correlation and validation**

- For each group, the spatial Pearson correlation between each pair of images is calculated
- Groups which contain features showing different spatial distribution, according to the desired correlation threshold, are marked

S4. Identification

- **DB manager:** Is a tool available in Pyxis installation. The DB manager allows to fragment or import *in silico* collections of compound structure (opensource or customized by the user, in .SDF format), and fragmentation is based on the FR-DB rules or experimental as well as predicted MS/MS spectra.
- **Pyxis Fragmentation Rules database (FR-DB):** For lipids there is a proprietary collection of fragmentation rules from literature and *in house* experimental data^1^. Proprietary or metabolite specific rules of fragmentation can be added by the user through the DB manager tool. Experimental MS/MS database can also be imported.
- **Identification color palette:** Identification results will be associated to a background color that provides hints on the reliability and the robustness of the result. Pyxis uses a traffic light color palette:
  - Dark Green: high-confidence identification; the compound molecular structure was successfully identified
  - Orange: identification awaiting approval; sum composition or compound class conflicts are present for the proposed identification
  - Red: not identified
- **Automated/semiautomated approval:** The approval of identification results can be automated or semi-automated. Indeed, in the Identification page, the user can set that all the green-labelled or 4-stars matches can be automatically approved. If more than one possible compound is proposed for a given *m/z* value (e.g. isobars, stereoisomers), the inspection of the proposed matches is required.
- **Match scoring:** In order to reduce the number of false positives, when the identification process based on the use of FR-DB is performed, Pyxis scores all the possible matches according to an index calculated as the weighted average of the following scores:
  - Mass score (SM): proximity of the experimental mass with the theoretical mass of the proposed match
  - Isotopic Pattern score (SIP): comparison of the experimental isotopic pattern abundance with the theoretical one of the proposed match (Isotopic Pattern Abundance score, SIPA) and comparison of the experimental isotopic pattern spacing with the theoretical one of the proposed match (Isotopic Pattern Spacing score, SIPS), combined as:

$$S_{IP}= \sqrt{S_{IPA}*S_{IPS}}$$

- Chaos score (CS): spatial distribution of the *m/z* density image

$$CS= 100*spatial chaos$$

- Fragment score (SF): evaluation of the number of fragments and relative intensity found (NM) in respect to the fragments theoretically predicted (NTOT) for the proposed match. In particular, if their *m/z* difference is below the tuneable error tolerance, that fragment ion will be included in the fragments score SF1 calculated as:

$$S_{F1}= 100*\frac{N_{M}}{N_{TOT}}$$

- An additional score that takes into account the ion intensity of the matched peak (Imatch) over the sum of the intensities of all the experimental peaks (Iexp) is also calculated as:

$$S_{F2}= 100*\frac{\sum I_{match}}{\sum I_{exp}}$$

The two scores are combined using the following function:

$$S_{F}= \sqrt{S_{F1}*S_{F2}}$$

- CCS score (SCCS): proximity of the experimental CCS with the theoretical CCS of the proposed match

The weight to be assigned to each score can be set by the user in the “Score” settings of the identification method. Usually, according to this overall score, the correct match is one of the two highest matches proposed. However, a fixed value for a reliable minimum score cannot be provided because strictly dependent on the instrument and experimental condition used and, above all, to the weights assigned to each score. The inspection of the proposed matches in order to assign the correct identification can be performed by manually checking each match and selecting the most reliable one. Alternatively, approval of the most reliable match can be automatically performed, by specifying whether the approved match should be the one with the highest overall score, and/or the one present in a given database, and/or the most frequently identified in all the samples. Once correct identifications have been approved, adducts of the same compound can be automatically found and merged in a unique identification, upon checking for same spatial distribution.

S5. Untargeted data analysis of MALDI-2 timsTOF data

S5.1: MALDI-TIMS-MSI of mouse kidney tissue import settings
- Data already centroid. Centroid spectra filtering threshold: 300

Mobilogram peak detection

- **Window**: 17
- **Degree**: 3
- **Iterations**: 3
- **Minimum SNR**: 1
- **Noise window size**: 0.1 amu
- **Minimum absolute intensity**: 100
- **Discard peaks under**: 1% of the base peak
- **Discard peaks with less than**: 3 points

Dataset building

- **m/z tolerance**: 0.02 amu
- **mobility tolerance**: 0.02
- **Minimum peak frequency**: 1%
- **Minimum peak intensity**: 500
- **Minimum spatial chaos**: 0.5
- **Isotopic clustering**: ON. (IP abundance deviation 10%; m/z image correlation threshold 0.1; max charge 1)
  S5.2: MALDI-TIMS-MSI of THP-1 derived macrophages import settings
- Data already centroid. Centroid spectra filtering threshold: 10

Mobilogram peak detection

- **Window**: 15
- **Degree**: 2
- **Iterations**: 2
- **Minimum SNR**: 1
- **Noise window size**: 0.1 amu
- **Minimum absolute intensity**: 0
- **Discard peaks under**: 1% of the base peak
- **Discard peaks with less than**: 3 points

Dataset building

- **m/z tolerance**: 0.02 amu
- **mobility tolerance**: 0.02
- **Minimum peak frequency**: 1%
- **Minimum peak intensity**: 100
- **Minimum spatial chaos**: 0.3
- **Isotopic clustering**: ON. (IP abundance deviation 10%; m/z image correlation threshold 0.1; max charge 1)
  Figure S5.1: MALDI-TIMS-MSI of mouse kidney tissue. Average mobilogram at *m/z* 790.53 shows two distinct features separated by ion mobility.

Figure S5.2: Average frame parameters allow focusing on specific regions of interest (ROIs) and improve visualization of low-abundance signals.
